# Fatty Acids in Membranes as Homeostatic, Metabolic and Nutritional Biomarkers: Recent Advancements in Analytics and Diagnostics

**DOI:** 10.3390/diagnostics7010001

**Published:** 2016-12-22

**Authors:** Carla Ferreri, Annalisa Masi, Anna Sansone, Giorgia Giacometti, Anna Vita Larocca, Georgia Menounou, Roberta Scanferlato, Silvia Tortorella, Domenico Rota, Marco Conti, Simone Deplano, Maria Louka, Anna Rosaria Maranini, Arianna Salati, Valentina Sunda, Chryssostomos Chatgilialoglu

**Affiliations:** 1 ISOF, Consiglio Nazionale delle Ricerche, 40129 Bologna, Italy; annalisa.masi@isof.cnr.it (A.M.); anna.sansone@isof.cnr.it (A.S.); giorgia.giacometti@isof.cnr.it (G.G.); annavita.larocca@isof.cnr.it (A.V.L.); georgia.menounou@isof.cnr.it (G.M.); roberta.scanferlato@isof.cnr.it (R.S.) silvia.tortorella@isof.cnr.it (S.T.); rotadomenico@gmail.com (D.R.); conti.marco89@gmail.com (M.C.); chrys@isof.cnr.it (C.C.); 2 Lipinutragen srl, Via di Corticella 181/4, 40128 Bologna, Italy; deplano@lipinutragen.it (S.D.); maria.louka@lipinutragen.it (M.L.); maranini@lipinutragen.it (A.R.M.); salati@lipinutragen.it (A.S.); sunda@lipinutragen.it (V.S.)

**Keywords:** sapienic acid, palmitoleic acid, membrane lipidomics, red blood cell membrane, fatty acid balance, gas chromatographic resolution, geometrical and positional isomers, membrane fatty acid biomarker

## Abstract

Fatty acids, as structural components of membranes and inflammation/anti-inflammatory mediators, have well-known protective and regulatory effects. They are studied as biomarkers of pathological conditions, as well as saturated and unsaturated hydrophobic moieties in membrane phospholipids that contribute to homeostasis and physiological functions. Lifestyle, nutrition, metabolism and stress—with an excess of radical and oxidative processes—cause fatty acid changes that are examined in the human body using blood lipids. Fatty acid-based membrane lipidomics represents a powerful diagnostic tool for assessing the quantity and quality of fatty acid constituents and also for the follow-up of the membrane fatty acid remodeling that is associated with different physiological and pathological conditions. This review focuses on fatty acid biomarkers with two examples of recent lipidomic research and health applications: (i) monounsaturated fatty acids and the analytical challenge offered by hexadecenoic fatty acids (C16:1); and (ii) the cohort of 10 fatty acids in phospholipids of red blood cell membranes and its connections to metabolic and nutritional status in healthy and diseased subjects.

## 1. Introduction

**Figure 1:**
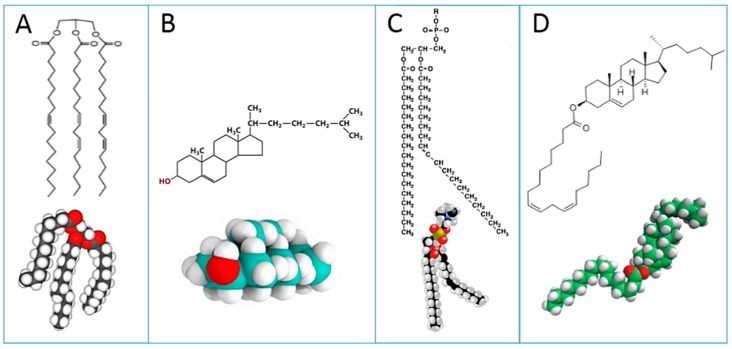
Main lipid classes present in blood: triglyceride (**A**); cholesterol (**B**); phospholipid as phosphatidyl choline (**C**); cholesteryl ester (**D**) shown as representative chemical structures and molecular models, showing the oxygen atoms in red and the hydrogen atoms in white, whereas the carbon atoms in the structures are in black or in other colors.

The last two decades have witnessed a strong interest in cell membrane phospholipids and fatty acid modulatory effects that are involved in the adaptability of living organisms. In particular, fatty acids have crucial roles in biophysical, biochemical and signaling processes that act as sensing mechanisms and stimuli transduction, thus participating in epigenetic control pathways [1,2,3]. Investigation of fatty acids and lipids for blood diagnostics started in the 1970s and nowadays this study has been taken over by lipidomics, implementing protocols and know-how toward new insights and applications [4,5]. Blood is easily withdrawn from living organisms in a non-invasive manner. Blood withdrawal is necessary for lipidomics, which has recently become the focus of studies that attempt to discern lipid diversity [4]. For non-expert readers, it is useful to see the main blood lipid classes in Figure 1: triglycerides (A); cholesterol (B); phospholipids (C); cholesteryl esters (D). Plasma lipoproteins are assemblies of the lipid molecules A–D with proteins.

These lipid classes show at a glance the relevance of lipids to fatty acids, whose distribution and metabolism is principally directed to provide the building blocks for cell membrane phospholipids of all tissues [5]. Analyses carried out on plasma give information about fatty acid dietary intakes of a few weeks before withdrawal, whereas blood cell membrane fatty acids (such as red blood cell, RBC) account for more stable information obtained from metabolic transformations together with stabilized dietary contributions. Mixtures of plasma and blood cells, such as a “blood drop”, have been proposed and discussed for medium-term dietary evaluation in population studies [6].

**Figure 2:**
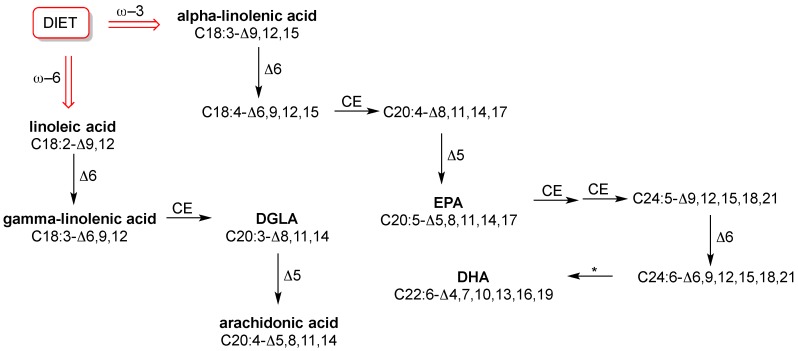
Major biosynthetic pathways of polyunsaturated fatty acids (ω-3 and ω-6) with the interplay of desaturation (Δ5, Δ6) providing double bonds in cis geometric configuration (vertical arrows). The elongation step adds a two carbon atom-unit to the fatty acid chain (CE, chain elonagtion). * beta-oxidation. DGLA: Dihomo-γ-linolenic acid; DHA: Docosahexaenoic acid; EPA: Eicosapentaenoic acid.

The structural diversity expressed by saturated, monounsaturated and polyunsaturated fatty acids (SFA, MUFA and PUFA, respectively) is able to provide a palette of arrangements for fine-tuning of membrane fluidity and permeability properties, as well as for receptor and channel functioning. Indeed, cell membrane functionality is strongly correlated with the balance reached by the specific quantities and qualities of fatty acids, in order to provide physiological responses and avoid pathological diversion [7,8]. However, in this balance the fact that humans do not biosynthesize all fatty acids plays a big role, because only SFA and MUFA are endogenously formed. PUFA components, with the wide diversity of their structures, influence fluidity and permeability in many ways and all human tissues need them. However, they are “essential”, which means that humans do not fabricate them. This is a trivial consideration; however, it can be seen that such basic knowledge is still not applied in practice. In fact, in routine check-up essential or semi-essential PUFAs are not considered. Neither the individual dietary intake of the omega-6 precursor linoleic acid (C18:2-Δ9,12) or the omega-3 precursor alpha-linolenic acid (C18:3-Δ9,12,15), nor the level of relevant fatty acids in cell membranes are established. Specific foods have to be taken regularly in order to ensure correct levels of these precursors and their transformations into long chain PUFAs, as shown in Figure 2. We will not explore this subject more deeply here, because it has been extensively treated in biochemistry books and also recent reviews [3,9].

With insufficient levels of PUFAs from the diet, essential fatty acid (EFA) deficiency occurs. This causes impairments, in particular at two levels: (i) structural and functional organization of membrane phospholipid assembly in tissues, such as omega-3 deficient retinal tissues [5]; and (ii) mediator and signaling activities, which starts from PUFA enzymatic detachment from membrane phospholipids and their release in cytoplasm, creating a balance among the resulting prostaglandins, leukotrienes, thromboxanes, resolvins, etc. [1,7,9]. All these processes are nowadays looked at with renewed attention, since they constitute the molecular basis of cell proliferation and degeneration, such as in inflammation and cancerogenesis [9,10,11,12,13].

Fatty acid-based functional lipidomics identifies fatty acid changes in the above-reported lipid classes of living organisms, and correlates results with physiological and pathological conditions, organizing an enormous amount of data for new perspectives in diagnostics and therapeutics. Depending on the lipid types, fatty acid-based functional lipidomics can involve membrane lipids, mediator follow-up or biomarker identification, and advancements of these branches have been summarized [3]. 

The purpose of this review is to discuss two aspects of recent research concerning fatty acids in membrane lipidomics. In this review we first consider the hexadecenoic fatty acid family (C16 MUFA), which represents a seminal example of analytical complexity in biological samples, in order to unambiguously determine position and geometry of the double bond along fatty acid chains with an equal length. This subject is strictly correlated to lipidomic protocols needed for biomarker development. Next, we describe the identification of a molecular cohort of fatty acids in erythrocyte membrane phospholipids, which expresses the homeostatic balance between saturated and unsaturated residues, and is needed for optimal function. This subject is strictly correlated to the scientific advance in understanding how metabolic and nutritional conditions influence the molecular balance in the cell membranes of each organism.

The relevance of the first focus on C16 monounsaturated fatty acids is due to their emerging role as biomarkers, as described in recent literature, and the need for an unambiguous recognition of the double bond position and geometry. It will be described how the challenge for the correct individuation of the C16 isomers was solved by protocols and molecular references that ensure analytical robustness for biomarker discovery.

The second focus concerns the role of fatty acids in the homeostatic balance of cell membrane evaluated by fatty acid-based membrane lipidomics. This involves utilizing a specific molecular cohort of fatty acids, of specific quantity and quality, which furnishes comprehensive information on the organization of this important biological compartment. We will describe how the composition of fatty acids in human red blood cell membranes has been translated into an effective comprehensive health biomarker that is increasingly adopted for the follow-up of nutritional and metabolic balance in healthy and diseased subjects.

The two subjects that are treated in this review will be of particular interest to specialists interested or involved in lipidomics and in the discussion of protocols for biomarker development, as well as those interested in research connected to cell signaling and response, who cannot disregard the importance of the fatty acid composition and remodeling in cell membranes. Finally, readers will understand how the influence of nutritional and metabolic status can be followed-up by fatty acid-based membrane lipidomics, adding more information to the protocols for prevention and disease management.

## 2. Hexadecenoic Fatty Acid Family as Biomarker in Blood

**Figure 3:**
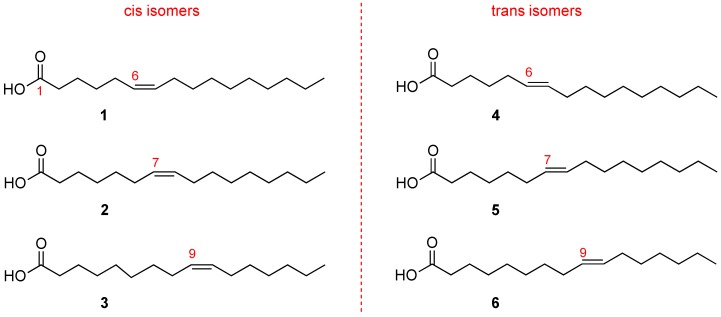
Positional isomers of hexadecenoic fatty acids with the carbonyl group in position 1 and the double bond in different positions along the chian numbered in red: in C6–C7 (**1**); C7–C8 (**2**) and C9–C10 (**3**). Each of these molecules can exist also as a geometrical trans isomer (**4**, **5** and **6**, respectively).

Hexadecenoic fatty acids are MUFAs that have a hydrocarbon chain with 16 carbon atoms (C16) and one double bond. In Figure 3 it is shown that the double bond can occupy different positions along the chain. Fatty acids with the same number of atoms, differing only in the double bond position along the chain, are called positional isomers.

The most popular positional isomer is the fatty acid with double bond in C9–C10, palmitoleic acid (C16:1-Δ9, **3** in Figure 3), which derives from palmitic acid transformation by stearoyl-Coenzyme A desaturase (SCD1). Sapienic acid (C16:1-Δ6, **1** in Figure 3) has the double bond in C6–C7, deriving from delta-6 desaturase (D6D) conversion of palmitic acid. The third positional isomer, i.e., with a double bond between C7–C8 (C16:1-Δ7, **2** in Figure 3) results from beta-oxidation of oleic acid.

The biological significance of C16 MUFAs has increased after the discovery that sapienic acid, formerly only connected to human sebum and bacteria metabolisms, is present in human blood lipids, lipoproteins and erythrocyte membranes [14]. More attention is now given to MUFA biosynthesis and metabolic transformations. Indeed, palmitic acid, which is known to be principally transformed into palmitoleic acid, was found to compete with linoleic acid for human delta-6 desaturase [15]. This indicated that the enzyme, known to work preferentially with PUFA, is able also to transform a saturated fatty acid. Delta-6 desaturase activity will certainly be a matter for further research, especially for its role when fatty acid synthase (FAS) enzyme and palmitic acid biosynthesis increase, as occurs in cancerogenesis [16].

At this point it is worth underlining that the C16 MUFA family also includes geometrical isomers (Figure 3) that have the same double bond position. However, the natural cis geometry changed into a trans configuration. The C16 MUFA isomers can be found in human tissues as well as in foods, like milk and cheese, and have already attracted attention for their analytical resolution [14,17]. So far, two trans isomers are known: the trans isomer of palmitoleic acid (C16:1-Δ9trans, called also palmitelaidic acid, **6** in Figure 3) and the trans isomer of sapienic acid (C16:1-Δ6trans, **4** in Figure 3), whereas the compound **5** (C16:1-Δ7trans, **see** Figure 3) has never been isolated from natural sources [17].

A brief digression on trans fatty acids (TFA) can be made at this point. TFAs are not biosynthesized by eukaryotes, and in 1999 it was discovered that the cis double bonds, ubiquitously present in natural fatty acids, are converted to the trans configuration by free radicals (usually, a sulfur-centered radical) [18,19]. TFAs endogenously formed by free radicals in living organisms are present in a small quantity (0.1%–0.4% in healthy human RBC [3]), highlighting a basal isomerizing free radical formation during metabolism. On the other hand, trans MUFA can be formed during bacterial biosynthesis, such as when bacteria are present in ruminant stomach. Therefore, a certain TFA quantity is contained in dairy products [17,18]. A third source of TFA is also an exogenous source that is present in processed fats, in particular TFA formed during the industrial oil processing of partial hydrogenation or deodorization [18].

Regarding the C16 MUFAs, palmitelaidic acid (**6** in Figure 3) is the main trans hexadecenoic isomer present in the human body and is connected with diet, as ascertained by a positive relationship with self-reported consumption of whole-fat dairy, butter, and margarine; it was also associated with higher LDL and cholesterol, but with lower triglycerides, fasting insulin, blood pressure, and incident diabetes in a multiethnic cohort in the United States [20]. Connection of trans isomers with health problems is reported in several reviews [17,18,21]. Endogenous formation of trans isomers associated with radical stress is distinguishable from exogenous contribution [18], and this is an important issue to address since these unnatural isomers are increased in diseases, similar to the way they are increased in obese patients compared to healthy controls [22,23,24].

As mentioned in the introduction, the C16 MUFA family represents a seminal example of analytical complexity in biological samples, in order to distinguish position and geometry of the double bond along fatty acid chains with an equal length. The protocol for ensuring analytical resolution of the isomers presented in Figure 3 is a preliminary issue in their development as a health biomarker. Gas chromatography (GC) is the gold standard for fatty acid isomer determination, and GC analysis is usually performed after transforming fatty acids obtained from biological samples into the corresponding fatty acid methyl esters (FAME) by mild methodologies [3,22]. Efforts have been made to choose appropriate oven conditions with long columns (60–100 m), coated with polar stationary phases such as cyanopropyl silicones or polyethylene glycols, to obtain good separation of positional and geometrical isomers, while keeping a reasonable time for GC runs. The observation that helium is better than hydrogen as carrier gas seems too technical, but it is important to know that carrier gas plays a role in the method of biomarker identification [14]. Column length information is also necessary to evaluate data reported in the literature on the presence of palmitoleic acid in biological samples after using GC analysis with a 30 m column and without any other positional isomer as a reference [25]. Knowledge about GC analysis, carrier gas and column length, together with expertise in mass spectrometry analysis, as detailed below, are necessary in view of the growth of lipidomics for biomarker development.

The recent study of the positional isomer C16:1-Δ7cis (**2** in Figure 3) as a possible biomarker of metabolic changes in disease onset, offers a case of analytical resolution. This isomer is contained in scarce quantities in dairy products; therefore, diet cannot be blamed for its contribution. It metabolically derives from oleic acid by a beta-oxidation step. Its level in human plasma phospholipids has been reported to be ca. 0.07%–0.1% [26,27]. Recently, a high level of this fatty acid was reported in human foamy monocytes, particularly in membrane phospholipids, suggesting its possible development as a biomarker for early detection of cardiovascular disease [28]. In this case, GC analysis was performed with dimethyloxazoline (DMOX) derivatives of cell membrane fatty acids, and in the GC run only one peak was detected for the delta-7 cis isomer superimposing with the delta-6 cis isomer (sapienic acid). Examining mass spectrometry (MS) data, mass fragments of pure reference compounds (sapienic acid and delta-7 isomers) were found to differentiate for the relative abundance of fragments at *m*/*z* = 167 and at *m*/*z* = 168, finding a higher abundance of the latter fragment for delta-7 isomer. Biological samples were then examined by GC/MS and by the abundance of the fragments relative to the unique C16 fatty acid peak. It was concluded that in the foamy monocytes only the delta-7 isomer was present, thus proposing its role as a biomarker of cardiovascular disease onset. 

**Figure 4:**
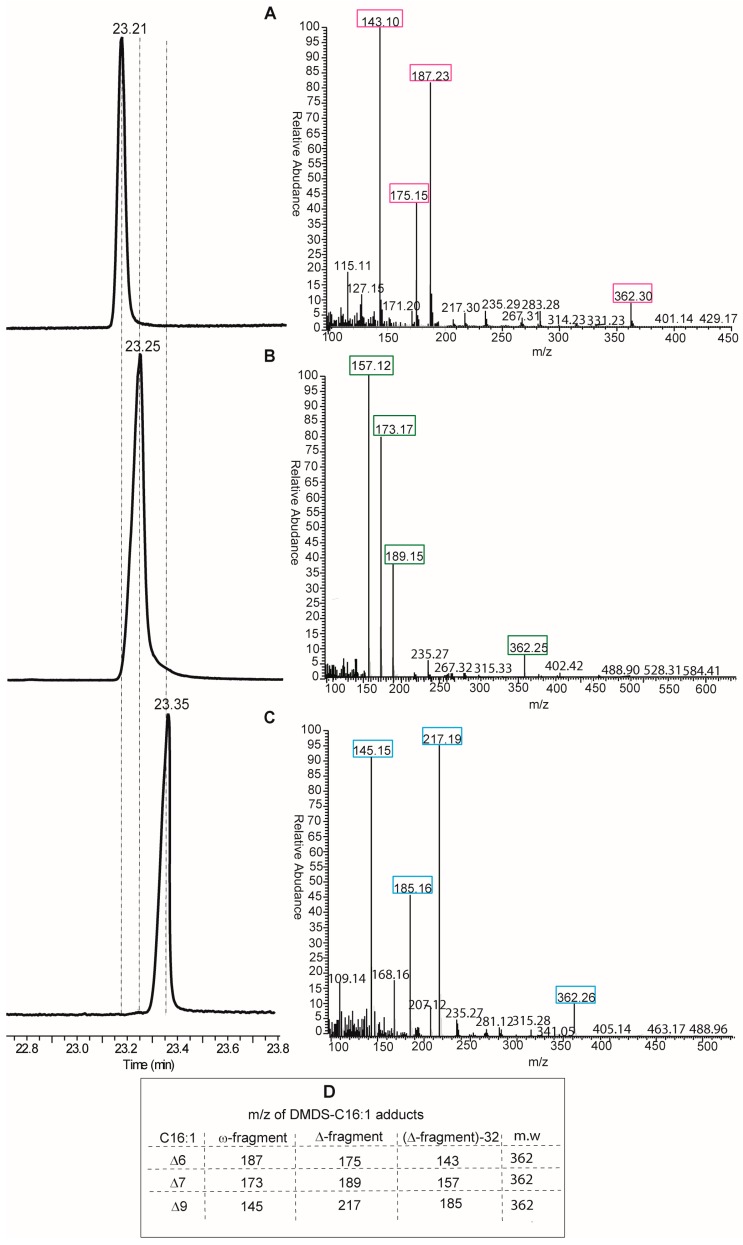
Gas chromatography (GC) elution window and mass spectra of the three dimethyl disulfide (DMDS) derivatives obtained from delta-6 (**A**); delta-7 (**B**) and delta-9 (**C**) with diagnostic mass fragments (ω-fragment and Δ-fragment) in colored boxes as well as in the panel **D** (see supplementary data of [14] for further details).

Looking at the protocols by GC and MS analyses, the DMOX derivatization of fatty acids is neither the only, nor the best, method for obtaining an unambiguous analytical resolution of positional and geometrical isomers. Indeed, dimethyl disulfide derivatives (DMDS) of FAME mixtures are extensively used for the analysis of biological samples of any kind. In particular, for the single double bond of MUFAs, this is the most direct method, since methyl sulfide substituents add exactly to the two carbon atoms of the double bond, thus affording different adducts depending on the positional isomer present in the mixture [14]. DMDS adducts can be also resolved by GC analysis because they have different elution times and, more importantly, because they give different fragments by mass spectrometry. In fact, fragmentation occurs at the level of the C–C bond where sulfide groups are attached, i.e., the double bond position previous to derivatization, and two fragments (called ω- and Δ-fragments) are formed (see Table in panel D, Figure 4). By their mass, the exact position of the double bond along the fatty acid chain is unambiguously obtained. An example of DMDS adducts of the three positional hexadecenoic isomers is shown in Figure 4, where GC runs are coupled with mass fragmentation [14]. It is easy to distinguish the diagnostic fragments of the DMDS adducts derived from the C16 MUFA isomers for their different masses (see Table in panel D, Figure 4), and not only by their abundance as in the case of DMOX derivatives. Therefore, we suggest that DMDS derivatization followed by GC/MS analysis is the protocol of choice in this biomarker identification. 

Such analysis, carried out on RBC membranes and cholesteryl esters, brought unequivocal identification of delta-6 and delta-9 positional isomers in humans, whereas the delta-7 isomer was below instrumental detection [22]. Examination of other lipid classes and tissues, such as triglycerides in human adipose tissues, preliminarily indicated that the presence of the delta-7 isomer, although in lower amounts than the delta-6 isomer, cannot be excluded [29].

It is worth mentioning that examination of lipid classes in the same subject (or sample) may attenuate the need to fully examine dietary and environmental influences. From the same blood sample, cholesteryl esters and RBC membrane phospholipids can be examined in parallel taking into account the fact that they can be formed, among other pathways, by the enzymatic activity of lecithin cholesterol acyl transferase (LCAT), transferring one of the two fatty acid tails of a phospholipid to cholesterol [30] (see Figure 1 for structures). The first example of measurement in these two lipid classes has been given in a recent work on morbidly obese patients [22], evidencing different trends of C16 MUFAs in the patients, compared to healthy subjects: sapienic acid increased in erythrocyte membranes and decreases in cholesteryl esters in patients, whereas palmitoleic acid increased in both lipid classes. Parallel evaluation of two lipid classes in the same individual can represent an important improvement of the diagnostic power of lipidomic analysis, especially when interfering factors from diet and lifestyle are not easy to control in patients and control groups.

## 3. Fatty Acid Cohort to Examine Membrane Balance and Human Profiles

The role of fatty acids, derived from biosynthesis and nutritional fats, as fundamental elements for fine regulation of membrane properties has been known for decades. Therefore, fatty acids cannot be disregarded when it comes to understanding how cells, tissues, organs and, finally, an entire organism work. In this view, not only is a single fatty acid or family relevant, but also the fatty acid assembly that unitarily forms membrane phospholipids, since it reaches a structural and functional balance, reflecting metabolic, environmental and dietary influences. This “holistic” view of membranes comes from multidisciplinary research in lipid biophysics, biology, and biochemistry, which is beautifully summarized by the definition of membranes as “metabolic pacemakers” [31].

This concept stems from a re-evaluation of the remodeling process known as Land’s cycle [32] that triggers, after stimulus, a reshaping of membrane fatty acid composition. For example, in the case of pancreatic beta cells, when increasing concentrations of their principal metabolite, glucose, are used, it was observed that membrane fatty acids change by diminishing omega-6 (linoleic and arachidonic acids) and omega-3 (docosahexaenoic acid, DHA) fatty acids. At the same time the MUFA content (palmitoleic and oleic acids) of membrane phospholipids increased. In this seminal paper, for the first time it was assessed that, in the metabolic response of pancreatic beta cells to glucose, the liberated omega-6 fatty acids in cytoplasm are connected with the occurrence of lipid peroxidation and formation of 4-hydroxynonenal (4-HNE), thus triggering insulin response by 4-HNE interaction with peroxisome proliferator–activated receptor-δ (PPAR-δ) [33].

Recently, research has been more intensely focused on cooperative mechanisms for membrane fatty acid reshaping, highlighting the role of lysophosphatidylcholine acyl transferase (LPCAT) enzymes [34,35]. Acyltransferase enzymes, that move fatty acid moieties from one lipid class to the other, together with fatty acid biosynthetic processes and nutritional availability of essential fatty acids, are all involved and contribute to membrane remodeling. It is worth underlining that fatty acid rearrangement in membrane phospholipids follows homeostatic principles. This means that membranes of each tissue cannot change ad libitum. Instead, phospholipid fatty acid diversity in membranes is a harmonized process, reaching a balance of SFA, MUFA and PUFA residues that respect the typical composition of each tissue [6]. This phenomenon guarantees the in vivo requirements for optimal membrane-associated processes, which are needed to cooperate with the genetic program of the specific cell type.

With these premises, in the last decade or so, research into membrane lipidomics has increased, and nowadays protocols are developed at cell, animal and human levels. Experimental conditions for cell membrane isolation, lipid extraction, phospholipid separation and analysis are reported in each work. As previously indicated, the identification of membrane fatty acids is mainly performed by transesterification of phospholipids to get FAME derivatives; using room temperature and methanolic alkaline conditions, ester-bound lipids are efficiently transformed into FAMEs. The typical and most diffuse membrane lipids are glycerophospholipids, in which the two fatty acid tails are esterified to glycerol, as shown in Figure 1 (structure C). As previously discussed, FAMEs obtained from membrane phospholipid derivatization are precisely identified by GC analysis coupled with MS.

One of the most extensive studies of the importance of dietary fats for membrane fatty acid composition was performed in rats, as a model of metabolism in response to behavior. Rats were fed 12 moderate-fat diets for 8 weeks. The diets differed only in the relative content of SFA, MUFA and PUFA components. Body tissues together with blood were obtained after the diet period, examining fatty acid contents [8]. The exhaustive fatty acid analysis of rat heart, brain, liver, RBC, and adipose tissues, as well as plasma, gave the opportunity to estimate the conformity of membranes to diet, an important parameter established in the study and impossible to obtain in living human subjects by withdrawing such a large variety of tissues. This study ascertained that tissue membranes are not strongly influenced by moderate fat intake, therefore they can be useful in examining metabolic influences. On the other hand, it was also importantly established that the composition of a tissue which has lost its typical fatty acid balance becomes more diet-responsive in order to recover from that imbalance. In this study RBC membranes are definitely proved as a homeostatically regulated tissue, containing SFA, MUFA, PUFA types, and others, and not being strongly sensitive to diet variation. Membrane phospholipid SFA content made up less than 50% of total fatty acids, with brain, liver and RBC having similar trends (ca. 45%). Also, MUFA levels did not strongly respond to diet with similar behavior in heart, liver, muscle and RBC. In this study, PUFA behavior to dietary contents was thoroughly investigated. Importantly, glycerophospholipids were found to be meaningful lipid classes to monitor PUFA changes expressed by the PUFA balance, i.e., the percentage of omega-3 over total PUFA. In all tissues, a PUFA balance of less than 10% corresponded to a better response to diet, evidencing that the level of essential fats in membranes responds to severe controls by cellular homeostasis and cannot go under a certain threshold [8]. PUFA contents in rats are highly variable in plasma triglycerides, as is also the case in humans. Indeed, to get information on stabilized nutritional conditions the blood cell such as erythrocytes are very informative, and cannot be substituted by analysis of serum or whole blood (mix of serum and blood cells).

Over the last 15 years, transfer of lipid science to study human physiological and pathological conditions has been the focus of much research, as expressed by two lipidomic platforms in Europe and the USA, namely, ELIFE [36] and LipidMaps [37], giving rise to databases of lipidomic expertise, standards and methods. Continuous research into the field will certainly bring more and more applications in molecular diagnostics. 

RBC membrane phospholipid composition in humans is considered very relevant information and, using fatty acid GC analysis, two purposes can be fulfilled: (a) to individuate each fatty acid as a biomarker of certain health conditions, as previously discussed for C16 MUFA isomers; and (b) to use a cohort of the most representative fatty acids in a membrane, enabling us to describe RBC membrane balance by a comprehensive indicator of homeostatic regulation [38,39]. Membrane lipids in RBC are strongly influenced by the previously mentioned remodeling and exchange processes. Since RBC loses its DNA quickly, biosynthesis of lipids and transcriptional processes cannot occur in this cell, which then expresses, better than others, its lipid homeostasis reached by circulating in the body and exchanging with all tissues.

For the purpose of defining the representative fatty acid cohort, the examination of published data from population studies and meta-analysis on healthy controls in different geographical location, age and sex [39,40,41,42,43] led to the individuation of a group of 10 fatty acids as ubiquitous components in RBC membranes that have well-known roles in metabolism and nutrition. They are: SFA with palmitic and stearic acids, MUFA with palmitoleic, oleic and vaccenic acids, and PUFA with the omega-6 linoleic, dihomo-gamma-linolenic (DGLA) and arachidonic acids and the omega-3 eicosapentaenoic (EPA) and docosahexaenoic (DHA) acids [3,39]. The structures of PUFAs are shown in Figure 2.

Taking into account that human RBCs have a mean lifetime of 120 days, and cell circulation and aging can influence membrane composition, a more specific cell sampling in the blood was performed, with a step of mature RBC selection before membrane isolation and fatty acid analysis. This choice increased the precision of the protocol and the resulting fatty acid analysis gives information on the stabilized membrane fatty acid balance reached in the mature RBC, which is close to its maximum lifetime (>3 months old) [44,45,46]. Mean values obtained for unselected RBCs and mature RBCs are summarized in Table 1 and reported as percent intervals of the fatty acid families. Further research is ongoing on mature RBC membranes and lipidome characterization, also using innovative biophotonic methodologies, recently discovered in our laboratories [47].

The analysis of membrane composition is interestingly connected with the lipid replacement therapy (LRP) proposed by Nicolson [48], the first discoverer of membranes as fluid mosaics. LRP pursues the replacement of membrane fatty acids, which can be consumed during continuous exposition of membranes to oxidative damage, with fresh ones given by an oral emulsion. It is evident that, estimating the fatty acid cohort from RBC membranes in each individual, the LRP approach could be easily personalized.

**Figure 5:**
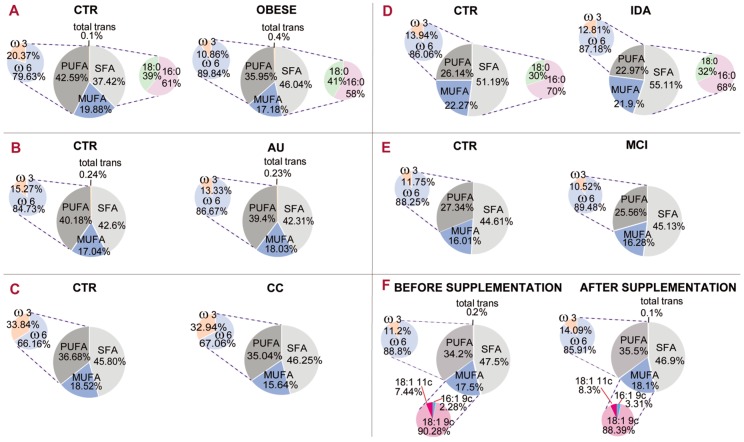
RBC membrane fatty acid families and types of human subjects of different ages in healthy (CTR) and pathological conditions (panels **A**–**E**); (**A**) morbid obesity [22]; (**B**) autism [44]; (**C**) colorectal cancer [49]; (**D**) iron deficient anemia [50]; (**E**) mild cognitive impairment [51]; panel (**F**) reports the RBC membrane status before and after an omega-3 supplementation of 3 months carried out in infertile men [45].

The cohort of membrane fatty acids was useful to rationalize data coming from different groups of human subjects and to represent their membrane balance among SFA, MUFA and PUFA, as previously discussed. Figure 5 gives an example of a comparative representation of data reported in the literature [22,44,45,49,50,51].

The membrane fatty acid cohorts in healthy and differently diseased subjects of Figure 5 can be discussed. As proposed for small mammals (rats) [8], PUFA balance in large mammals (such as humans) can give a measure of homeostatic, nutritional and metabolic contributions, and can be easily calculated from the results reported in published human studies. As discussed above, in rats it was found that a PUFA balance <10% (indicating a homeostatically de-regulated condition) rendered the tissue more sensitive to diet. By using published human studies, the fatty acid distributions in different health conditions are reported in Figure 5. It is gratifying to see that the PUFA balance (pie chart representation on the left of each panel) is in the 10%–12% interval in several diseased conditions, such as obesity (panel A, 10.86%), mild cognitive impairment (panel D, 10.52%) and infertile men (panel F, 11.2%). In one case of mild cognitive impairment (panel D), the PUFA balance in healthy controls is 11.75%, indicating a low PUFA balance also in this cohort, but the difference with patients is also evident (10.52%). It must be underlined that in the literature, work on PUFA levels has already led to the individuation of two indices: the ω-6/ω-3 ratio [52] and the omega-3 index, the latter established in RBC membranes as cardiovascular risk index [53]. According to the state-of-art research reported so far, it seems acceptable to introduce the PUFA balance of 10%–12% as a low limit value for humans that can be ameliorated with personalized nutritional strategies. It can be foreseen that implementation of a mathematical relationship using the values of fatty acids and families will provide important comprehensive health biomarkers and expand lipid molecular tools for health and disease evaluation.

Certainly, more investigations and data surveys are necessary to define all components of the “healthy balance” for humans, but in this respect membrane fatty acids, chosen for their quantity and quality, look very promising. It could also be suggested to systematically introduce the mature RBC membrane fatty acid cohort and PUFA balance values in prevention plans for healthy people, in order to cover this important molecular aspect influencing the basal metabolism and tissue functioning. Finally, it can be extrapolated that nutraceutical supplementation or dietary treatment in people with low PUFA balance would be more efficiently driven due to the tissue homeostatic requirement. The importance of these concepts for studies concerning the effects of nutritional treatments on membrane lipid composition can be envisaged. In fact, one of the reported cases in Figure 5 (panel F) shows that supplementation of nutritional quantities of omega-3 fatty acids in infertile men led to an amelioration of PUFA balance from 11.2% to 14.09% [45].

## 4. Conclusions

In conclusion, two aspects concerning the development of biomarkers in blood, i.e., the analytical protocol for fatty acid isomer recognition and the RBC membrane fatty acid cohort as a comprehensive health biomarker, have been presented. Very precise analytical protocols and an elaboration of data available in the literature have been discussed, suggesting further applications in molecular diagnostics for evaluation of nutritional and metabolic status. The discussion revealed the need for multidisciplinary teams in lipidomics, covering the aspects of biomarker development ranging from chemistry and analytics to biochemistry, molecular biology, nutrition and medicine. 

Finally, the membrane fatty acid cohort and PUFA balance represents assessed scientific knowledge that is ready to be used as non-expensive molecular tools in routine check-up and population monitoring. This will provide relevant information on healthy conditions and help to evaluate the effects of nutritionally-based strategies.

## Figures and Tables

**Table 1:** Fatty acid families of membrane phospholipids obtained from human healthy red blood cell (RBC) and healthy mature RBC.

Fatty Acid Family	RBC (%) ^§, a^	Mature RBC (%) ^#, a^
SFA	40.6–49.8	34–45
MUFA	14.4–20.3	15–23
PUFA omega-6	27.4–34.0	24–34
PUFA omega-3	3.2–7.8	5.7–9
Total PUFA	32.2–40.2	30–43

^§^ values adapted from [38]; ^#^ values adapted from [3,41,44,45,46]; ^a^ reported as % of each fatty acid family taking into account a cohort composed of the following fatty acids: SFA (palmitic and stearic acids), MUFA (palmitoleic, oleic and vaccenic acids) and PUFA (omega-6: linoleic, dihomo-gamma-linolenic and arachidonic acids; omega-3: eicosapentaenoic and docosahexaenoic acids).
